# Update on Rome IV Criteria for Colorectal Disorders: Implications for Clinical Practice

**DOI:** 10.1007/s11894-017-0554-0

**Published:** 2017-04-03

**Authors:** Magnus Simren, Olafur S. Palsson, William E. Whitehead

**Affiliations:** 1 Department of Internal Medicine & Clinical Nutrition, Institute of Medicine, Sahlgrenska Academy, University of Gothenburg, 40530 Gothenburg, Sweden; 2 Center for Functional Gastrointestinal and Motility Disorders, University of North Carolina at Chapel Hill, Chapel Hill, NC USA

**Keywords:** Functional GI disorders, Rome IV diagnostic criteria, Functional bowel disorders, Irritable bowel syndrome, Functional anorectal disorders, Fecal incontinence

## Abstract

**Purpose of Review:**

The purpose of the review was to provide an update of the Rome IV criteria for colorectal disorders with implications for clinical practice.

**Recent Findings:**

The Rome diagnostic criteria are expert consensus criteria for diagnosing functional gastrointestinal disorders (FGIDs). The current version, Rome IV, was released in May of 2016 after Rome III had been in effect for a decade. It is the collective product of committees that included more than 100 leading functional GI experts. For functional bowel and anorectal disorders, the majority of changes relative to Rome III are relatively minor and will have little impact on clinical practice. However, notable changes with potential impact on clinical practice and research include the changes in the diagnostic criteria for IBS, the modified approach for subtyping of IBS, the view on functional bowel disorders as a spectrum of disorders, and the new definition of fecal incontinence.

**Summary:**

New features in the Rome IV diagnostic criteria for functional bowel and anorectal disorders will likely have modest influence on clinical practice, with a few exceptions.

## Introduction

In May of 2016, the new diagnostic criteria for functional gastrointestinal disorders, the Rome IV criteria, were published [1]. In this review, we will provide an update on the functional bowel and anorectal disorders as defined in Rome IV (Tables 1 and 2) [2••, 3••] and highlight differences relative to Rome III [4, 5], including how these changes might influence clinical practice and future research.

**Table 1:** Rome IV—Bowel Disorders (2)

1.Irritable bowel syndrome (IBS)
a.IBS with predominant constipation (IBS-C)
b.IBS with predominant diarrhea (IBS-D)
c.IBS with mixed bowel habits (IBS-M)
d.IBS unclassified (IBS-U)
2.Functional constipation
3.Functional diarrhea
4.Functional abdominal bloating/distension
5.Unspecified functional bowel disorder
6.Opioid-induced constipation

**Table 2:** Rome IV—Anorectal Disorders (3)

1.Fecal incontinence
2.Functional anorectal pain
a.Levator ani syndrome
b.Unspecified functional anorectal pain
c.Proctalgia fugax
3.Functional defecation disorders
a.Inadequate defecatory propulsion
b.Dyssynergic defecation

## Overview of Rome IV

The Rome diagnostic criteria are expert consensus criteria for diagnosing functional gastrointestinal disorders (FGIDs) that have been in use in the field of gastroenterology for more than a quarter of a century, since their first iteration in 1990 [6]. Over this time period, understanding of the disorders has steadily evolved, and it has therefore been necessary to update the criteria periodically to bring them into alignment with new research findings and understanding of symptom patterns and pathophysiological contributing factors. The criteria have evolved through four successive versions. In each instance, the updates have reflected years of hard work by teams of experts in each of the five major functional GI domains (esophageal, gastroduodenal, bowel, biliary, and anorectal disorders), who are tasked with updating the criteria and writing the associated documentation to ensure that the criteria are in tune with the latest knowledge in the field [7••]. The current version, Rome IV, was released in May of 2016 [1] after Rome III had been in effect for a decade. It is the collective product of committees that included more than 100 leading functional GI experts. Although the new diagnostic criteria themselves are the backbone of Rome IV, the accompanying books and key scientific articles written by the Rome committee members can also be considered an important part of the update. These publications provide important reviews and reference materials on the disorders, including comprehensive overviews of the basic and clinical literature on the disorders, updated treatment algorithms, and new relevant information on gut microbiota, brain-gut interactions, physiology, pharmacogenomics, psychosocial and cultural factors, symptom subgroups within the disorders, and potential biomarkers.

## Innovations that Distinguish Rome IV from Rome III

The group of GI diagnoses found in Rome IV is broadly similar to Rome III [7••]. Except for four new diagnoses that were added in this version (reflux hypersensitivity, cannabinoid hyperemesis syndrome, opioid-induced constipation, and narcotic bowel syndrome/opioid-induced GI hyperalgesia), the changes from Rome III consist for the most part of relatively minor adjustments in diagnostic requirements. Both clinicians and researchers who have relied on earlier Rome criteria will therefore generally find the disorders defined by Rome IV criteria to be very familiar. In nearly all cases, the key sets of symptoms are the same as before.

Beyond changes in content of the diagnostic criteria, however, Rome IV also involved innovations in the Rome process and philosophy. These consisted of increased empirical grounding of the criteria as well as a partial shift in the terminology used to describe the disorders.

## Empirically Informed Frequency Thresholds

A significant refinement in the approach of the Rome committees in the Rome IV work was a conscious effort to make the symptom frequency thresholds for the diagnoses evidence based. For this purpose, a nationwide US population-based internet survey of 1665 individuals stratified by sex, age, and race, was conducted specifically for the Rome Foundation [8••]. The survey included questions on all the Rome III symptoms with new symptom frequency response scales that had been devised for the Rome IV diagnostic questionnaire; for most symptoms, this was either a percentage scale or a more detailed absolute-frequency scale than previously used. The resulting report provided the Rome committees with frequency distributions of symptoms in the general population, which aided them in selecting cut-offs for the new criteria that were most likely to separate healthy individuals from those with abnormal symptoms. In general, the recommended standard was that diagnostic thresholds for individual symptoms should incorporate less than 10% of the general non-clinical population (i.e., when all individuals with relevant GI disorders were excluded from the analysis). This empirically informed approach led to changes in the required symptom frequencies for several diagnoses in comparison to Rome III. The new Rome IV criteria for IBS are perhaps the most notable example of this, as will be discussed below.

## Validation of the Diagnostic Criteria Prior to Publication

Another Rome IV refinement of the process of developing the criteria was a much more detailed validation of the criteria ahead of their official release than was done for earlier Rome versions. This was accomplished by conducting a nine-site study [8••] in the USA, UK, and Canada of 843 adult gastroenterology clinic patients with established functional GI diagnoses (these three countries with English-speaking populations were selected for the test because the diagnostic questionnaire was only available in English before publication). Participating patients completed the Rome IV diagnostic questionnaire and the agreement between clinical diagnoses and diagnoses according to the Rome IV criteria was examined. The main focus was on three of the most common FGIDs: irritable bowel syndrome (IBS), functional constipation (FC), and functional dyspepsia (FD). Diagnostic sensitivity was moderate for IBS (62.7%) and dyspepsia (54.7%). However, functional constipation by Rome IV only showed modest sensitivity (32.2%). The explanation for the low sensitivity for that disorder seems to have been that clinicians tended to ignore the Rome stipulation of excluding people who meet IBS (and in Rome IV, also OIC) criteria from the FC diagnosis. This is supported by the fact that follow-up analyses in the study found that sensitivity was much better (72.2%) if exclusion of IBS and OIC was not required for FC diagnosis. The same study also assessed a 1-month test-retest reliability of the new Rome criteria in the clinics and found the same diagnoses were reproducible in about 75% of patients, which seems quite reasonable considering that many of the patients were receiving new treatments for their key diagnostic symptoms in the meantime.

In parallel to this nine-site clinical validation study, a population survey of 5931 adults in the same three countries was conducted as a part of the validation process. Those survey data were used to evaluate the specificity of the Rome IV criteria, i.e., their ability to accurately exclude non-patients in the general population from the diagnoses. The specificity of the Rome IV criteria was found to be excellent for all the Rome IV diagnoses that could be assessed with confidence (including 97.1% for IBS, 93.6% for FC, and 93.3% for FD).

## Transition from Functional GI Disorders to Disorders of Brain-Gut Interaction

The Rome IV criteria and their associated publications reflect a philosophical change in the conceptualization of the family of GI disorders they cover through reduced emphasis on the term “functional” in their nomenclature. In recent years, there has been growing recognition that multiple specific pathophysiological processes play a role in functional GI disorders, including imbalance between different types of gut bacteria, increased gut permeability, and altered immune function. Furthermore, the importance of neural and hormonal interaction between the brain and the gut in producing and modulating the symptoms of the disorders has been recognized. For these reasons, it has increasingly seemed to many thought leaders in the field that applying the functional descriptor to these disorders is overly simplistic or misleading and can promote stigmatization by fostering the inaccurate notion that they have no real physical causes. However, it was also recognized by the committees working on Rome IV that the term functional gastrointestinal disorders has become a widely recognized and broadly used name for this entire class of health problems and that sudden abandonment of the word functional in the nomenclature of these disorders might cause confusion. For this reason, a strategy of gradual shift was adopted instead. The official Rome IV publications by the Rome Foundation offer the alternative and preferred term “Disorders of Gut-Brain Interaction” as a new umbrella term for functional gastrointestinal disorders, while the older term is used in those publications as well. Furthermore, the word functional has been omitted in some names for individual diagnoses and groups of diagnoses. For example, functional esophageal disorders are now simply called esophageal disorders, functional fecal incontinence is now simply fecal incontinence, and functional abdominal pain syndrome has been renamed centrally mediated abdominal pain syndrome. It is likely that this gradual phasing out of the descriptor functional in referring to these gut-brain disorders will continue in future work of the Rome committees.

## What Is New in the Bowel Disorders in Rome IV?

### Classification

Several recent studies have highlighted the substantial overlap between various functional bowel disorders, as well as substantial flux between these disorders and between the IBS subtypes [9–11, 12••, 13, 14, 15••]. Therefore, in Rome IV it is emphasized that functional bowel disorders constitute a spectrum of GI disorders rather than isolated entities. It is acknowledged that, even though they are characterized as distinct disorders based on diagnostic criteria, significant overlap exists, and occasionally, it may be difficult to distinguish them as distinct entities. Furthermore, it is also highlighted that transition from one functional bowel disorder to another, or from one predominant symptom to another, is frequently seen, and this may occur as part of the natural course of the disorder, as a response to therapy, or both. Another new feature in the Rome IV classification of bowel disorders is a new disease category, opioid-induced constipation (OIC), which will be described in greater detail below.

## Irritable Bowel Syndrome

Compared with Rome III [5], there are two major changes in the IBS diagnostic criteria in Rome IV [2••]. The first is that abdominal discomfort has been deleted from the definition, which means that abdominal pain is now mandatory to make the diagnosis of IBS. In Rome III, the IBS definition was “…abdominal pain *or discomfort* associated with defecation or a change in bowel habit…” [5], but in Rome IV the definition is “…abdominal pain associated with defecation or a change in bowel habits…” [2••]. This change was based on the imprecise nature of the term “discomfort,” and the fact that “discomfort” is not present in every language or has different meanings in different languages. Furthermore, it is not clear if the distinction between discomfort and pain is qualitative or quantitative, that the understanding of the meaning of discomfort varies substantially between individuals, and that it is considered to encompass a wide range of symptoms [16]. The other major change was the change in the symptom frequency threshold. In Rome IV, abdominal pain should be present at least 1 day per week on average during the preceding month, compared with at least 3 days per month in Rome III. This was based on data obtained in the Rome normative GI symptom survey [8••]. Both of these changes are likely to reduce the prevalence of IBS in population-based studies, but will probably not affect the prevalence of IBS in clinical populations to any major degree, since most patients with IBS acknowledge pain as one of their main symptoms [16], and the majority of IBS patients in clinical samples have symptoms more frequently than once per week [17]. Furthermore, slight rewording of the IBS main criteria was also included in Rome IV, so that the abdominal pain should be “…*related* to defecation…” (Rome IV), instead of “…*improved* with defecation…” (Rome III), to emphasize the fact that a substantial proportion of IBS patients actually reports worsening of pain with defecation [18, 19], and/or associated with a change in stool frequency and/or stool form. It is also acknowledged more clearly in Rome IV compared with Rome III that the diagnosis of IBS should be made following several steps using a combination of the diagnostic criteria and a limited number of additional tests based on the presenting symptoms (the same strategy recommended for all functional bowel disorders).

In Rome III, IBS patients were classified into one of four subtypes based on proportion of bowel movements with abnormal stool consistency as defined in the Bristol Stool Form (BSF) scale (types 1 and 2: hard, lumpy stool; types 6 and 7: loose, watery stools) [20, 21]; IBS with constipation (IBS-C), IBS with diarrhea (IBS-D), IBS with mixed bowel habits (IBS-M), or unsubtyped IBS (IBS-U) [5]. However, studies using stool diaries with the proposed cut-off for defining the subgroups (25% hard and/or loose stools according to BSF) resulted in a very large proportion of subjects being defined as IBS-U, and a very small proportion fulfilled criteria for IBS-M [22–24], since IBS patients frequently have days with bowel movements with normal stool consistency (BSF3-5) [12••, 17]. Moreover, when using the proposed cut-off retrospectively in diagnostic questionnaires (“sometimes hard and/or loose stools”), an exceedingly high proportion instead were categorized as IBS-M [25]. Based on these shortcomings with Rome III IBS subtyping, a new approach was suggested in Rome IV, but with the same subtypes, i.e., IBS-C, IBS-D, IBS-M, and IBS-U (renamed “unclassified” instead of “unsubtyped”) [2••]. In clinical trials, 2-week stool diaries [26] using the BSF scale is recommended, but the predominant bowel habit for subtyping is based on stool form on days *with at least one abnormal bowel movement* (BSF 1–2 or 6–7), i.e., days with only bowel movement with normal stool consistency, BSF 3-5, are not counted. The 25% cut-off for defining the subtypes is also used in Rome IV: IBS-C: >25% hard stools and <25% loose stools; IBS-D: >25% loose stools and <25% hard stools; IBS-M: >25% loose stools and >25% hard stools; and IBS-U: <25% loose stools and <25% hard stools. This approach will most likely substantially reduce the IBS-U group, and increase the IBS-M group, which reflects experience from clinical practice. As an alternative for epidemiology or clinical practice, subjects can be shown a picture of the BSF scale and asked to define if their abnormal bowel movements are usually constipation (BSF 1-2; IBS-C), usually diarrhea (BSF 6-7; IBS-D), both constipation and diarrhea (IBS-M), or not applicable since they rarely have abnormal bowel movements (IBS-U).

## Functional Constipation, Functional Diarrhea and Functional Abdominal Bloating/Distension

For these disorders, there were minor changes in Rome IV [2••] vs. Rome III [5]. The most notable change was that for these disorders, where the wording “…there are insufficient criteria for IBS…” is part of the diagnostic criteria, it is now recognized that mild pain and/or bloating may be present, but are not predominant symptoms in functional constipation and functional diarrhea, and that mild pain related to bloating as well as minor bowel movement abnormalities may be present in functional abdominal bloating/distension. These changes are in line with the idea that functional bowel disorders should be thought of as existing on a continuum rather than as discrete disorders, i.e., being a spectrum of chronic GI disorders with combinations of symptoms attributable to the lower GI tract.

For functional diarrhea, the cut-off was changed from 75% of stools being loose in Rome III to >25% in Rome IV, but just like in Rome III, it was acknowledged that a thorough clinical work-up is needed before this diagnosis should be made. For practical reasons, the 25% threshold for the symptoms included in the diagnostic criteria for functional constipation (straining, lumpy or hard stools, sensation of incomplete evacuation, sensation of anorectal blockage, use of manual maneuvers to facilitate defecation, and fewer than three spontaneous bowel movement per week) was kept in the Rome IV criteria, even though slightly different cut-offs are probably optimal for epidemiological studies based on findings in the Rome normative GI symptom survey [8••].

## Opioid-Induced Constipation

Significant advances have been made in the area of opioid-induced bowel dysfunction in recent years, including development of new therapies and better understanding of underlying mechanisms and differences between the various constellations of symptoms related to the intake of opioids [27–29]. Even though traditionally not consistent with the definition of a functional GI disorder (“underlying cause not known / well defined”), it was decided to include OIC among the functional bowel disorders in Rome IV, based on the frequent overlap and shared underlying mechanisms. OIC is defined in Rome IV as new or worsening symptoms of constipation when initiating, changing, or increasing opioid therapy, that must include two or more of the symptoms defining functional constipation (see above) with the same frequency cut-off (25%). Furthermore, narcotic bowel syndrome or opioid-induced GI hyperalgesia, characterized by paradoxical development of, or increase in, abdominal pain associated with continuous or increasing dosages of opioids, was also included in Centrally Mediated Disorders of GI Pain in Rome IV, a diagnostic category which was also new relative to Rome III [30••]. Given the increasing use of opioids in the US and in other parts of the world, these entities will most likely be more commonly seen in clinical practice in the coming years, and important to manage appropriately [31]. Hence, these updates in the diagnostic criteria seem clinically important.

## What Is New in the Anorectal Disorders in Rome IV?

### Fecal Incontinence

The diagnostic criteria for fecal incontinence underwent substantial changes between Rome III and Rome IV: Rome III attempted to distinguish Functional Fecal Incontinence from structural or neurogenic causes [4] whereas Rome IV defines Fecal Incontinence (FI; not functional fecal incontinence) simply as the uncontrolled passage of solid or liquid stool with no distinction made on the basis of presumed etiology [3••]. This reflects an emerging consensus that there are multiple, overlapping factors that contribute to the occurrence of FI and that patients for whom the causes of FI are primarily psychological or related to bowel habit abnormalities are rare. Moreover, the distinction between functional and structural or neurological causes has not been found to be a reliable guide to effective treatment. Although this is a major conceptual change in the way the Rome criteria define FI, it will not have a great impact on clinical practice because most clinicians already diagnose and treat based on the assumption that there is no clear distinction between functional and structural causes of FI.

A second change in the diagnostic criteria for FI is to require, at least for research purposes, that stool leakage occur at least two times in a 4-week period to qualify for the diagnosis. By contrast, Rome III required only “recurrent” (i.e., more than one) episodes of stool leakage in the past 3 months. This more restrictive frequency threshold in Rome IV will result in fewer patients with mild or infrequent stool leakage meeting the criteria for FI and may result in lower estimates of the prevalence of FI. Research is needed to determine whether this frequency threshold reliably distinguishes between patients with infrequent and mild stool leakage who have less quality of life impairment and are satisfied to manage on their own vs. patients with more frequent stool leakage who may benefit from treatments available through a physician.

## Rectal Pain Syndromes

Changes from Rome III to Rome IV in the diagnostic classification of functional anorectal pain syndromes are relatively few and are unlikely to have a significant impact on clinical practice:

The term “chronic proctalgia” was dropped from the classification system. The Rome III criteria [4] distinguished two major types of anorectal pain: chronic proctalgia and proctalgia fugax, based on differences in the quality of pain (dull pain or aching in chronic proctalgia vs. sharp or stabbing pain in proctalgia fugax) and duration of pain episodes (chronic pain present most of the time or for periods greater than 20 min in chronic proctalgia vs. fleeting, brief pain episodes (20 min or less) separated by pain-free periods in proctalgia fugax). In Rome III, chronic proctalgia was further subdivided into levator ani syndrome if traction on the levator muscles during digital rectal examination elicited a report of tenderness or pain, vs. unspecified functional anorectal pain if such digital traction did not elicit a report of tenderness. However, a factor analysis study [32] of a population sample of young adults did not identify distinct clusters of symptoms for chronic proctalgia vs. proctalgia fugax, so the term “chronic proctalgia” was eliminated from the classification system in Rome IV [3••]. Nevertheless, because the pathophysiological mechanisms and treatment indications for these two disorders may differ, the distinction between levator ani syndrome, unspecified functional anorectal pain, and proctalgia fugax were preserved in Rome IV.

The maximum duration of pain episodes separating proctalgia fugax from levator ani syndrome and unspecified functional anorectal pain was changed from 20 min to 30 min based on anecdotal evidence that proctalgia fugax “attacks” sometimes last up to 30 min. Moreover, the location of pain in proctalgia fugax is the rectum in Rome IV; it was described as “lower rectum or anus” in Rome III.

The most significant innovation in the area of rectal pain syndromes relates to treatment and new insights into the pathophysiology of pain in levator ani syndrome, based on results from a randomized controlled trial comparing the three most commonly employed treatment strategies for chronic proctalgia (biofeedback, electrical stimulation of the anal canal, and massage of the levator muscles) [33••]. This study demonstrated that biofeedback (a protocol identical to the one used for correcting dyssynergic defecation) is the preferred treatment, but if biofeedback is not available, electrical stimulation may also be beneficial. Furthermore, the best predictor of the success of biofeedback treatment is a digital rectal exam showing tenderness with traction on the striated levator ani muscles, and the physiological mechanisms associated with clinical improvements in levator ani syndrome were acquisition of the ability to relax pelvic floor muscles when attempting to evacuate and acquisition of the ability to evacuate a water-filled balloon. These findings suggest similarities in the pathophysiological mechanisms between levator ani syndrome and pelvic floor dyssynergia even though the patients in this study did not report symptoms of constipation.

## Functional Defecation Disorders

The Rome IV working team introduced a number of changes to the diagnostic criteria for functional defecation disorders which are listed below. However, these are unlikely to significantly change which patients receive a diagnosis of functional defecation disorder or the indications for treatment.

The Rome III criteria required that the patient must meet symptom criteria for Functional Constipation. However, in deference to new data showing a strong overlap between functional constipation and irritable bowel syndrome with constipation (IBS-C) [15••, 34, 35], the Rome IV criteria state that the patient may fulfill this criterion by meeting symptom criteria for either functional constipation or IBS-C.

As with Rome III, the criteria for functional defecation disorders also require the presence of at least two of three objective physiological tests, but the nature of the three qualifying tests has changed: In Rome III, the first criterion was evidence of impaired evacuation by either an abnormal balloon evacuation test or imaging. In Rome IV, these have been separated; abnormal balloon expulsion is one criterion and an imaging study showing inadequate evacuation is a separate qualifying criterion. The Rome III list of qualifying criteria included inadequate rectal propulsive force, but this has been dropped from Rome IV. The third qualifying criterion is now described as an abnormal anorectal evacuation pattern assessed by anal manometry or surface EMG but, unlike Rome III, there is no specific definition of what an abnormal evacuation pattern is.

Both Rome IV and Rome III criteria define Inadequate Defecatory Propulsion and Dyssynergic Defecation as subtypes of Functional Defecation Disorders, and the criteria for this distinction are identical in Rome III and Rome IV.

Although the diagnostic criteria for functional defecation disorders remain largely the same in Rome IV, there is an emerging controversy in the field which may have an impact on clinical practice: Over the past 5–10 years, high-resolution/high-definition anorectal manometry equipment has been more widely disseminated, at least in academic medical centers. The expectation was that these systems would improve the diagnostic accuracy of anorectal manometry because the amount of data reported out is more than an order of magnitude greater [36]. However, two different research teams have found that more than 80% of healthy controls show evidence of paradoxical contraction or failure to relax the pelvic floor muscles when performing simulated defecation maneuvers [37••, 38]. This is inconsistent with earlier reports of a low frequency of paradoxical contraction in healthy subjects when perfusion manometry or manometry catheters with only a few solid state pressure transducers were used, and it calls into question the whole concept of dyssynergic defecation and the criteria that have been used to make this diagnosis. These observations also challenge the basis for pelvic floor biofeedback to treat disordered defecation despite multiple randomized controlled trials showing biofeedback training for pelvic floor relaxation to be a highly effective treatment for patients with difficulty evacuating the rectum [39–41]. The outcome of this controversy will inevitably have a significant impact on the standards for diagnosis and management of functional defecation disorders.

## Conclusion

In this review, we have provided an update on the most recent diagnostic criteria for functional bowel and anorectal disorders, the Rome IV criteria [2••, 3••]. The majority of changes relative to Rome III [4, 5] are relatively minor and will have little impact on clinical practice. However, notable changes with potential impact on clinical practice and research include the changes in the diagnostic criteria for IBS, the modified approach for subtyping of IBS, the new perspective on functional bowel disorders as a spectrum of disorders, and the new definition of fecal incontinence that makes no distinction based on presumed etiology. Furthermore, a new diagnostic entity, opioid-induced constipation, was included among the functional bowel disorders in Rome IV.
